# Challenges and Triumphs in Enterocutaneous Fistula Management: A Case Report of Complex Surgical Intervention and Multidisciplinary Care

**DOI:** 10.7759/cureus.67743

**Published:** 2024-08-25

**Authors:** Abhinav C G, Sachin Sholapur, Shirish Bhagvat, Akanksha A Barnwal, Manarth D Chhowala

**Affiliations:** 1 General Surgery, Grant Government Medical College and Sir JJ Group of Hospitals, Mumbai, IND

**Keywords:** advanced wound care, enteral and parenteral nutrition, temporary abdominal closure, surgical complication, surgical nutrition, enterocutaneous fisulae, entero-atmospheric fistula

## Abstract

Enterocutaneous fistulas (ECF) present complex challenges following abdominal surgery, involving abnormal communication between the gastrointestinal system and skin. We report an intriguing case of a 50-year-old female with a history of appendiceal perforation, primarily managed by right hemicolectomy with ileotransverse anastomosis, which led to an anastomotic leak and eventually an ECF. Failed conservative management, prompting re-exploratory laparotomy revealing extensive adhesions and iatrogenic enterotomies secondary to attempted adhesiolysis, led to multiple fistulae, further complicated by failed abdominal closure leading to a large abdominal wound to be managed along with the numerous enteroatmospheric fistulae.

Our comprehensive, structured approach included surgical care, nutritional support, and meticulous wound management, emphasizing patient comfort and recovery. If there were a graphical representation of the patient's smile and hope during the hospital stay, there would be a remarkable upward trend, symbolizing recovery and resilience. This case underscores the critical decisions and multidisciplinary teamwork required for the successful management of severe ECF, emphasizing holistic, patient-centered care.

ECF is one such field that has been well researched in the medical literature, but what makes this case report special is the multifaceted management of a case complicated at all three phases of surgical management, that is, preoperative, intraoperative, and postoperative.

## Introduction

An enterocutaneous fistula (ECF) is an abnormal connection between the stomach, small or large bowel, and the skin that allows gastrointestinal contents to flow onto the skin [1]. According to the literature, ECF is caused by various reasons, the most prevalent of which is surgical complications [2]. Other risk factors include malignancy, inflammatory bowel disease, post-radiation therapy for cancer, distal blockage, iatrogenic or spontaneous intestinal damage, complex intra-abdominal infections such as tuberculosis, amoebiasis, and typhoid, and diverticular disease [3]. We show an intriguing example of complex EC fistulas and their problematic care.

The patient, a 50-year-old female with a history of controlled hypertension, underwent an exploratory laparotomy and right hemicolectomy for acute appendicitis complicated by perforation, leading to subsequent complications. She presented with persistent fecal discharge from the abdominal drain and suture line, severe diffuse abdominal pain, dyspnea, and fever. Imaging confirmed an enterocutaneous fistula and peritonitis due to an anastomotic leak. Despite conservative management initially, worsening symptoms necessitated re-exploratory laparotomy on postoperative day 26, followed by intensive care management. Gradual improvement was noted with the stabilization of vital signs, the transition from parenteral to enteral nutrition (EN), and wound care using a wound manager device. At discharge on postoperative day 72, the patient had healed abdominal wounds with a stoma bag in place, planning for elective surgery to manage residual enteroatmospheric fistulae. Monthly follow-ups showed sustained recovery, with the patient's weight returning to preoperative levels.

## Case presentation

Patient background and chief complaints

A 50-year-old female with a history of controlled hypertension was referred to our center from a private hospital following exploratory laparotomy and right hemicolectomy with ileo-transverse anastomosis performed 25 days ago for acute appendicitis complicated by perforation of the base of the appendix and cecum. Despite initial surgical intervention, she developed persistent issues, including fecal discharge from both the abdominal drain and suture line, ongoing severe abdominal pain unresponsive to analgesics for the past three days, and a midline wound dehiscence with associated stool discharge. These symptoms were accompanied by progressive dyspnea over the last two days and fever with chills noted for the past two days, prompting further evaluation and management.

Physical examination and findings

**Figure 1:**
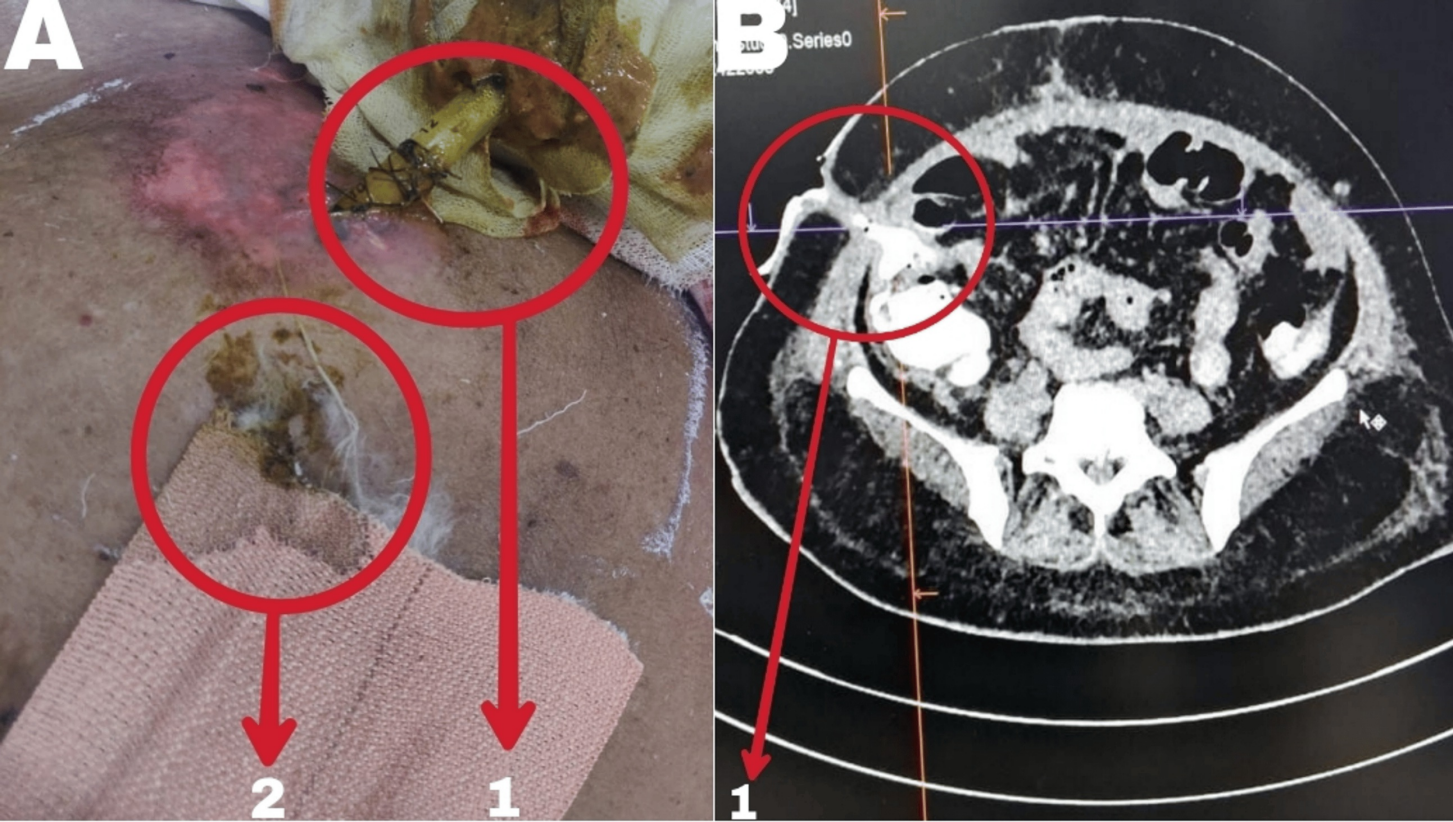
A) Fecal discharge from drain site and suture line on presentation; B) Contrast-enhanced computed tomography abdomen plus pelvis (CECT A+P) on presentation A1: fecal discharge from abdominal drain site with skin excoriation around drain site; A2: Fecal discharge from suture line with soaked dressing B: CECT (A+P) showing complete anastomotic dehiscence and contrast extravasation from ileal loops to drain site, suggestive of enterocutaneous fistula (5.5 mm thick, 35 mm long). Multiple intra-abdominal collections, indicative of peritonitis secondary to anastomotic leak B1: Enterocutaneous fistula (5.5 mm thick, 35 mm long)

On examination, the patient appears conscious, cooperative, and oriented to time, place, and person. Vital signs reveal a pulse rate of 124 bpm, blood pressure of 130/88 mm Hg, and oxygen saturation of 94% on room air, improving to 99% with supplemental oxygen at 6 L via face mask. Respiratory examination indicates decreased breath sounds with bilateral crepitations over the left lung fields. Abdominal examination reveals diffuse tenderness without guarding or rigidity, audible bowel sounds, and evident fecal discharge from the drain and suture line (Figure 1A). A midline wound dehiscence of 6x4 cm with a rectus breach further complicates the clinical presentation, necessitating urgent intervention and multidisciplinary management. Laboratory findings on presentation have been summarized in Table 1.

**Table 1:** Laboratory investigations on presentation Reference: [4]

Parameter	Value	Reference range
Hemoglobin	11.5 g/dL	Male: 14-18 g/dL, female: 12-16 g/dL
Total leukocyte count (TLC)	23,000 cells/mm³	4,000-11,000 cells/mm³
Neutrophils	90% of TLC	50-70%
Potassium (K⁺)	3.8 mmol/L	3.5-5.0 mmol/L
Albumin	3 g/dL	3.5-5.5 g/dL
Arterial blood gas (ABG) analysis on room air		
pH	7.41	7.38-7.44
HCO_3⁻_ (bicarbonate)	25 mmol/L	23-26 mmol/L
pCO_2 _(partial pressure of CO_2_)	60 mmHg	38-42 mmHg
pO_2 _(partial pressure of O_2_)	70 mmHg	75-100 mmHg (at sea level)

Contrast-enhanced CT (CECT) abdomen and pelvis

Midline wound dehiscence (6x4 cm) with communication to abdominal collections. Complete anastomotic dehiscence and contrast extravasation from ileal loops to the drain site suggest an enterocutaneous fistula (5.5 mm thick, 35 mm long). Multiple intra-abdominal collections are indicative of peritonitis secondary to an anastomotic leak (Figure 1B).

Postoperative course timeline

Postoperative Day (POD)

POD 0 is the day of exploration and right hemicolectomy with ileo-transverse anastomosis in view of perforation at the base of the appendix and cecum. A telephonic conversation with the operating surgeon from the private hospital revealed that there was a perforation at the base of the appendix and adjoining cecum, and thus the decision to go ahead with the right hemicolectomy was made. The distal end of the ileum, about 5-10 cm from the ileocecal junction, was anastomosed end to end with the transverse colon, about 3-4 cm from the hepatic flexure, in four layers. The abdominal drain was placed at the site of the anastomosis.

POD 1-4: The patient was passing stools and flatus and was started on a diet on POD 3.

POD 5-7: The patient complained of abdominal distension, pain, discharge from the suture site, and fecal output from the drain. Managed conservatively with nil per os (NPO), broad-spectrum intravenous (IV) antibiotics, and monitoring of drain output.

POD 8-20: Fecal output via drain averaged around 200-250 cc/day. Continued conservative management with NPO and IV antibiotics.

POD 22: The patient complained of severe acute-onset diffuse abdominal pain, worsening fecal discharge from the drain (>500 cc/day), and difficulty breathing.

POD 25: Referred to higher center for further management due to worsening clinical condition. Presented with ongoing complaints of stool discharge from drain and suture site, severe abdominal pain, difficulty breathing, and fever with chills. Broad-spectrum intravenous antibiotics targeting enteric pathogens to manage suspected peritonitis and wound infection were started. Total parenteral nutrition (TPN) providing 50% of estimated calorie requirements was initiated. Deep vein thrombosis (DVT) prophylaxis with compression stockings was also started.

Calculation for Nutritional Requirements

The body mass index of the patient upon presentation was 20.3 kilograms (kg) per square meter, with a weight of 52 kg, a height of 162 centimeters (cm), and an age of 50 years.

**Figure 2:**
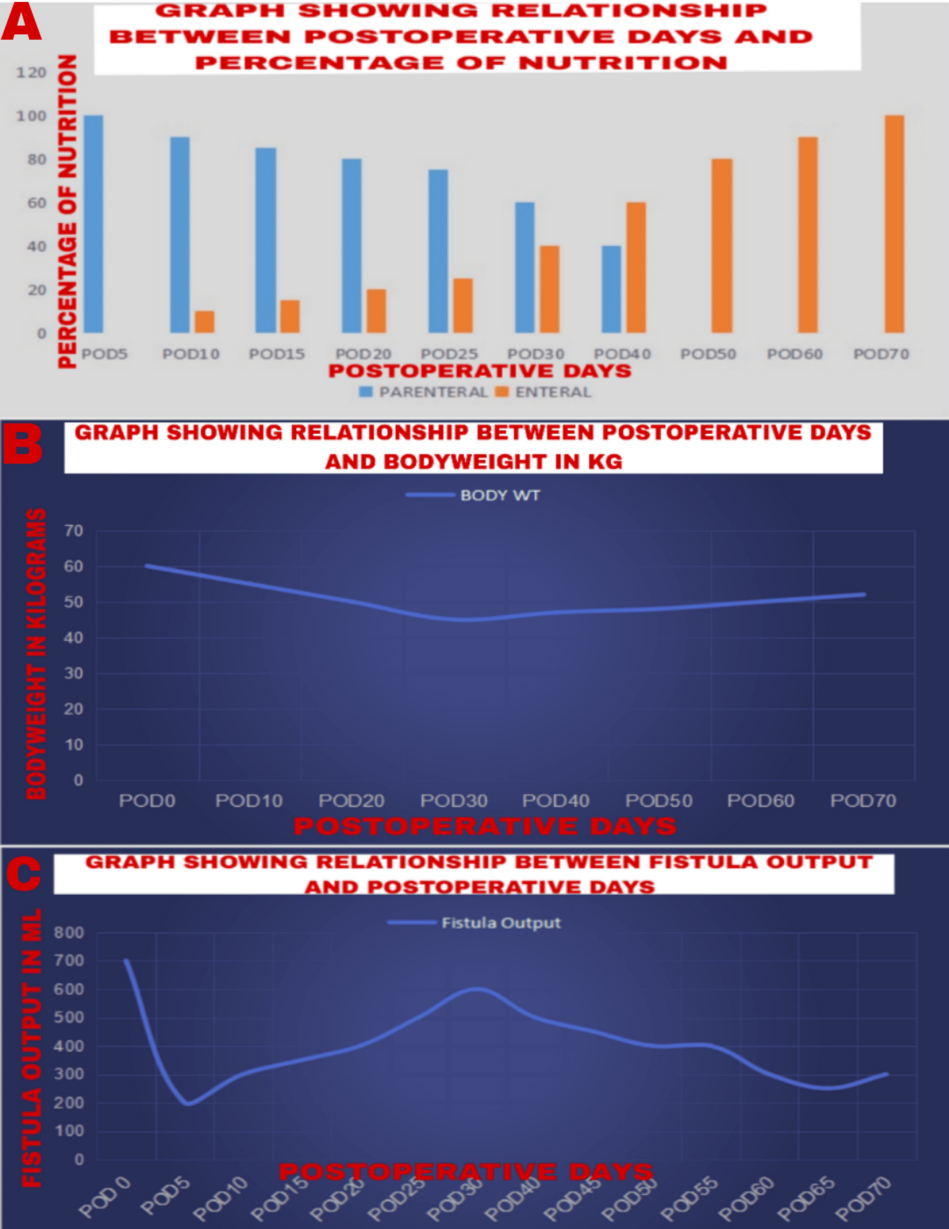
A) Graphical representation of shift from parenteral to enteral nutrition; B) Graphical representation of body weight of patient; and C) Graphical representation of fistula output during hospital stay

Daily energy requirement [5] and protein requirement were calculated applying ASPEN FELANPE guidelines [6]. Following permissive underfeeding, nutrition supplementation was started with 50% of the calculated requirements, initially with parenteral nutrition (PN) and gradually starting with EN as and when tolerated by the patient (Figure 2A) [7]. Changes in body weight and fistula output with respect to postoperative days after nutritional management have been depicted graphically in Figure 2B and Figure 2C, respectively.

**Figure 3:**
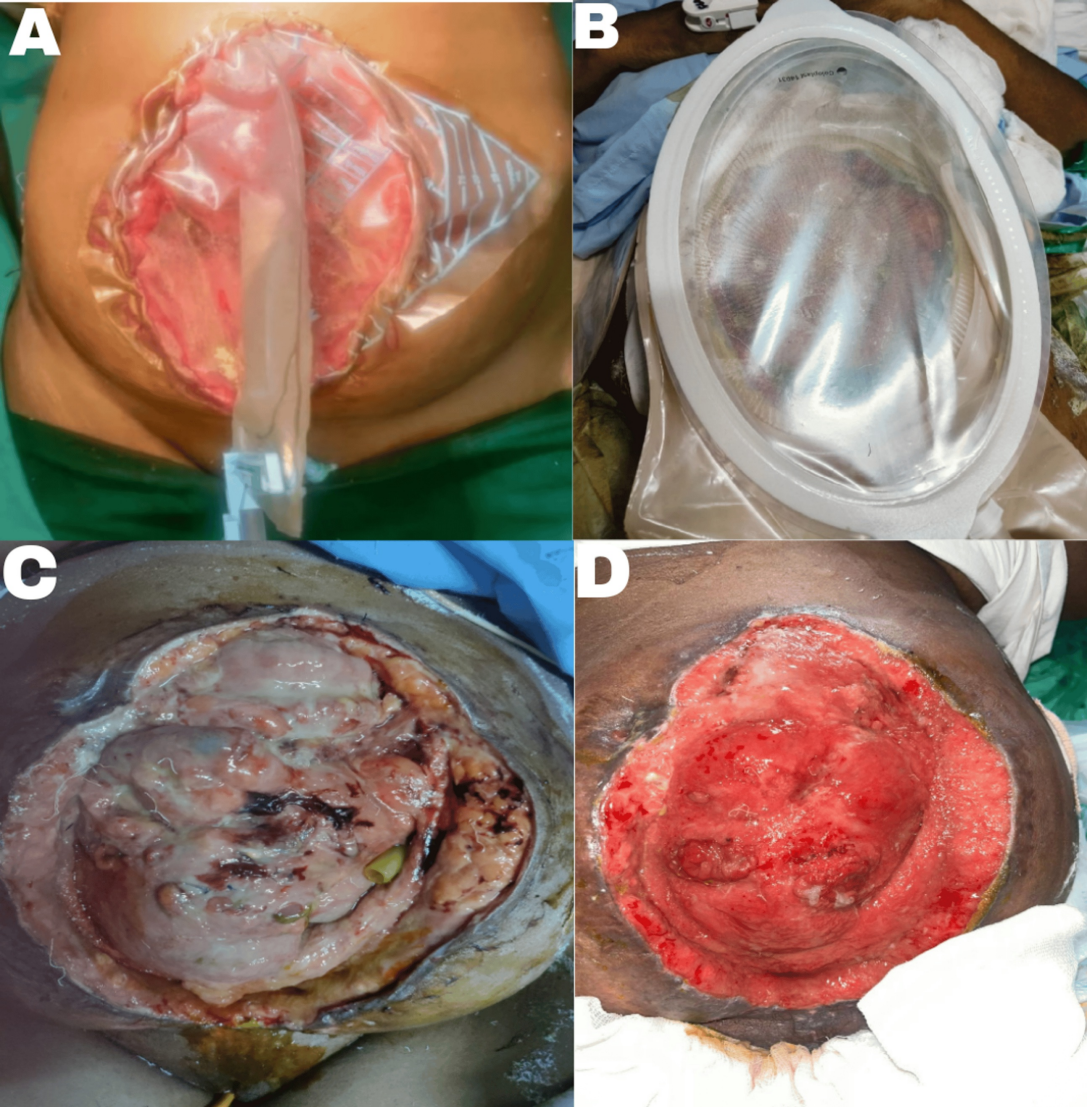
Comparison of abdominal wound at various stages during the postoperative course A: Application of Bogota bag post-re-exploration on POD 26; B: Application of wound manager for the large abdominal wound on POD 30; C: Before the first application of wound manager on POD 30; D: After the first application of wound manager POD 38 POD: postoperative day

POD 26: A re-exploratory laparotomy was performed due to suspected peritonitis and high-output enterocutaneous fistula. Extensive adhesions were encountered, and attempted adhesiolysis with two small enterotomies closed primarily. Intra-abdominal lavage and replacement of abdominal drains were performed. All attempts for abdominal closure failed and thus Bogotá bag was placed (Figure 3). Managed post-surgery in the critical care unit (CCU) with ventilation and inotropic support initially.

POD 28: Extubated on POD 28 and stable enough to be transferred out of CCU. Gradual improvement was noted with the stabilization of vital signs and weaning off ionotropic support and supplemental oxygen.

**Figure 4:**
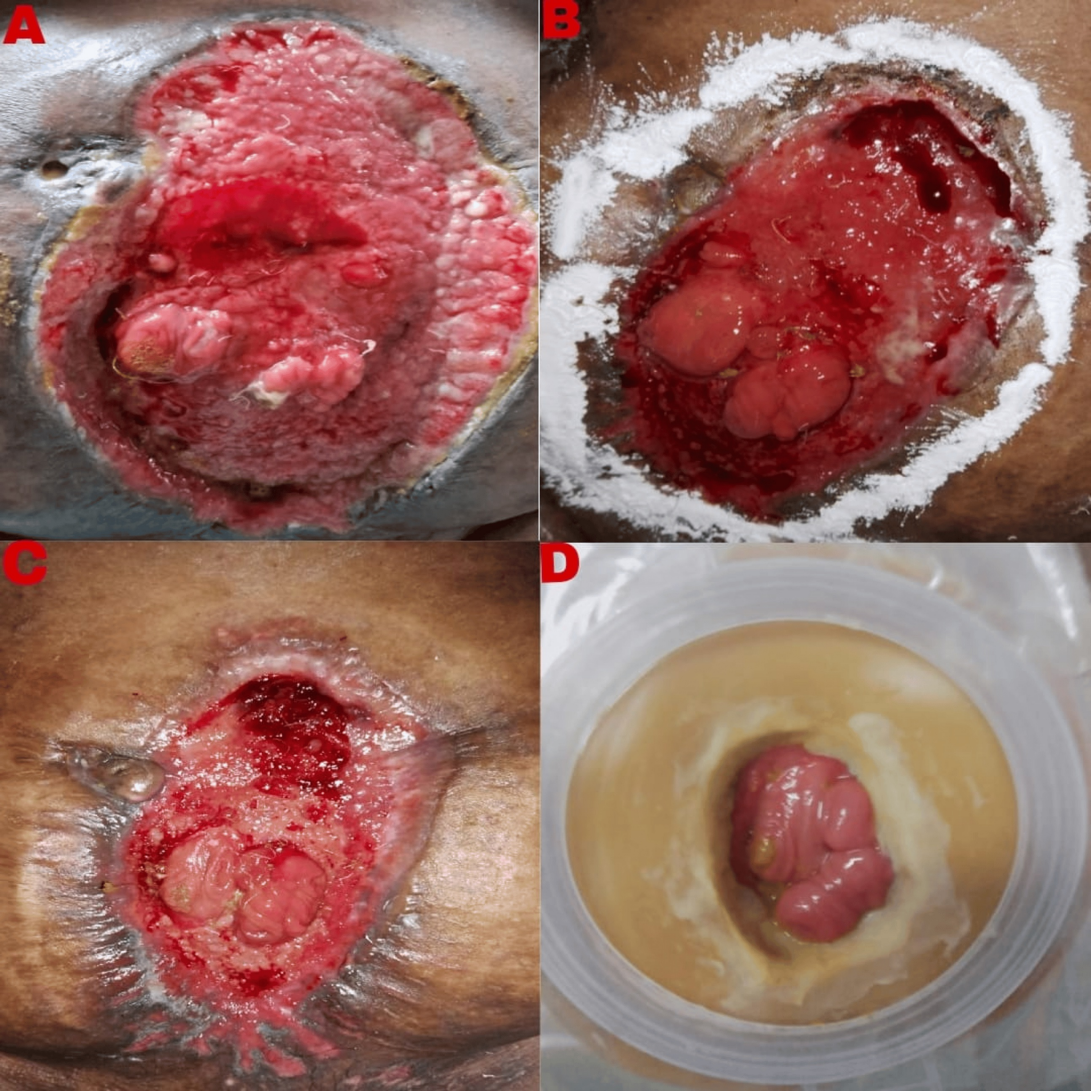
Comparison of abdominal wound from POD 46 to 132 A: After the second application of wound manager POD 46; B: After the third application of wound manager POD 54; C: After the fourth application of wound manager POD 70; D: After 60 days post-discharge, POD 132 This picture shows that the entire abdominal wound has healed with two loops seen inside the well-placed stoma bag, which are most probably distal ileal loops. POD: postoperative day

POD 30-60: Continued management with wound care using a wound manager device for midline wound dehiscence. Gradual improvement was noted with the stabilization of vital signs and weaning off ionotropic support and supplemental oxygen. Transitioned from PN to EN as tolerated. Ongoing monitoring of fistula output (Figure 2A), nutritional status, and wound healing (Figures 4A-4D).

POD 70: Imaging (CECT) showed two enteroatmospheric fistulae involving ileal loops. Continued multidisciplinary management focusing on nutritional support, monitoring the progress (Figure 2B), wound care, and respiratory rehabilitation.

POD 72: The patient was discharged on a full diet with a diet plan according to nutritional requirements along with supplementation of micronutrients.

Follow Up

The patient was followed up on a monthly basis for six months. Currently, six months after discharge, the patient has a stoma bag with the rest of the abdominal wound completely healed with two openings, proximal and distal, both being the distal ileum (Figure 4E).

Further Plan of Management

Take down the enteroatmospheric fistulae as a definitive elective surgery as the patient’s overall health is currently in a good state. The current weight of the patient is 60 kg which was her preoperative weight.

## Discussion

The exact frequency of enterocutaneous fistula remains unknown. Surgical causes, in most cases, are often dependent on the expertise of the surgeon, the health and immune status of the patient, details of the surgery, and the treated underlying condition. For example, Teixeira et al. reported in 2009 that ECF did develop in approximately 1.5% of patients after laparotomy [8]. Earlier, Tsuei et al. reported in 2004 that fistula occurred in 16.9% of cases managed by laparotomy for conditions such as gastrointestinal sepsis, pancreatitis, or trauma [9].

The formation of spontaneous fistula related to inflammatory bowel disease, like Crohn's disease, has also not been well explored. Tang et al. found in 2006 that 22.1% of 1595 patients with Crohn's disease developed fistulas [10]. Apart from surgical causes, spontaneous fistulas have been described in 23% to 48% of cases due to conditions like Crohn's disease and diverticulitis [10].

Classification

There are several classifications of enterocutaneous fistulas (ECF) [11], including categorization by output, etiology, and source. Typically, high-output ECFs are defined as exceeding 500 mL in 24 hours, low-output as less than 200 mL in 24 hours, and moderate-output fistulas fall between 200 and 500 mL in 24 hours. ECFs are also classified by their organ of origin: type I (abdominal, esophageal, gastroduodenal), type II (small bowel), type III (large bowel), and type IV (enteroatmospheric, irrespective of origin).

Management

Our approach to the management of enterocutaneous fistula is based on the three-phase strategy of Schecter et al. [12]. The first phase is recognition and stabilization. Our main objective after detecting an ECF is to stabilize the patient as quickly as possible. This phase is vital in respect of fluid and electrolyte management, nutrition, control of sepsis, abscess prevention, and control of wound infections. These issues need to be addressed within a time frame of 24-48 hours after identification of the fistula to prevent morbid conditions associated with mortality [3,12].

The subsequent phase involves defining the anatomical characteristics and making decisions based on diagnostic procedures such as fistulogram and CT scans. Endoscopy may also be utilized as needed. A fistulogram is particularly valuable for precisely locating and measuring the fistula, assessing whether it's single or multiple, and determining its proximity to anatomical landmarks like the pylorus, ileocecum, or anus. CT scans help identify abdominal abscesses and potential intestinal obstructions, enabling timely intervention through procedures like percutaneous drainage [3,12]. Understanding the anatomy of the fistula informs decisions regarding the potential for spontaneous closure, with surgical intervention considered if conservative management fails to yield results within 4-6 weeks [12].

A commonly used protocol in ECF management is encapsulated by the acronym "SNAP," emphasizing the importance of addressing skin care, managing sepsis, ensuring adequate nutrition, and precisely defining the fistula anatomy to guide treatment decisions [13].

Historically, traditional management of ECF has involved TPN while minimizing EN to reduce fistula output. TPN has demonstrated efficacy in reducing gastrointestinal secretions, which is crucial for managing high-output fistulas. Introduced in the 1970s, TPN significantly improved the nutritional status, closure rates, and survival rates of patients with fistulas. It plays a pivotal role in reversing the catabolic state associated with ECF, providing a window for spontaneous healing of fistulas that persist despite initial management efforts. For those requiring surgical closure, TPN facilitates infection control and enhances the likelihood of successful outcomes [14].

Enteral feeding has been shown to promote gastrointestinal mucosal health. Compared to TPN, EN lowers infection risks and associated expenses. Many facilities employ a mix of PN and EN. At the Nanjing Fistula Treatment Center of Jingling Hospital, out of 1,168 patients, 75.9% received combined PN and EN, while 13.6% received PN exclusively. The overall recovery rate was 93% [15].

The phrase "If the gut works, use it or prepare to lose it" [16] emphasizes the importance of utilizing the gastrointestinal system when feasible. In critically ill surgical patients, EN offers significant advantages over PN. EN is believed to enhance intestinal barrier function, decrease infection rates in critically ill patients, and sustain immune function more effectively than PN. Additionally, EN has been identified as a standalone factor linked to fistula closure [15,16].

According to the ASPEN-FELANPE Clinical Guidelines, EN can be suitable and well-tolerated for patients with low-output ECFs, defined as producing less than 500 ml/day, provided there is no distal obstruction. However, patients with high-output fistulas (more than 500 ml/day) may require PN to adequately meet their fluid, electrolyte, and nutritional needs [6,17].

The mortality rate is notably higher in patients with high-output ECFs, reaching approximately 30%, compared to around 6% for those with low-output fistulas [18]. Enteroatmospheric fistulas (EAFs), which occur in open abdomens, present significant challenges for nutritional support. There is limited research specifically on the use of EN for EAFs. Generally, early initiation of EN is recommended as the initial nutritional strategy for EAF patients, unless it could exacerbate shock or severe intestinal blockage. Starting EN early has been successfully implemented in EAF patients following open abdomen procedures, leading to improved survival rates [19].

Complications

ECFs are linked with serious health challenges and potential loss of life. Common issues arising from ECFs include infections, malnutrition, and imbalances in electrolytes and fluids. While less frequent, intestinal failure is a severe complication that carries substantial risks and can lead to significant health decline and death. These complications are the primary reasons for fatalities among individuals affected by ECFs [18,19].

Limitation

This is a single case report related to a field that already has well-established extensive medical research. Thus, a multicentric structured research study with robust data would help to draw a more valuable conclusion and add value to the already existing medical literature.

## Conclusions

This case report highlights intricate challenges encountered in managing a severe enterocutaneous fistula arising from complex surgical complications. Upon presentation, the patient exhibited distressing symptoms including persistent fecal discharge, abdominal pain, and respiratory difficulties, necessitating a comprehensive, multidisciplinary approach. Initial conservative measures proved insufficient, leading to the development of peritonitis and subsequent re-exploratory laparotomy revealing extensive adhesions and multiple fistulae. The management strategy involved the use of a Bogota bag for wound management, meticulous wound care, and tailored nutritional supplementation. Throughout the postoperative course, close monitoring of fistula output, nutritional status, and wound healing enabled timely adjustments in treatment strategies, facilitating a successful transition from TPN to enteral feeding.

The collaborative efforts of surgical specialists, critical care teams, and nutritionists played a pivotal role in stabilizing the patient and promoting recovery. The case underscores the importance of a structured, multidisciplinary approach in not only overcoming immediate surgical challenges but also in enhancing long-term outcomes and quality of life for patients. Ultimately, the patient's journey from initial presentation to discharge reflects a significant improvement in clinical status, symbolizing the profound impact of dedicated healthcare management in restoring hope and well-being amidst complex medical conditions.
